# Correction: Allosteric coupling in ligand-gated ion channels

**DOI:** 10.1085/jgp.20121084408242026c

**Published:** 2026-10-12

**Authors:** David Colquhoun, Remigijus Lape

Vol. 140, No. 6 | https://doi.org/10.1085/jgp.201210844 | November 26, 2012

The authors are grateful to Claudio Grosman (University of Illinois Urbana-Champaign, Urbana, IL, USA) for drawing their attention to errors in the labelling of Fig. 6. Fig. 6 B and errors in Table 1 and the text have been corrected. These corrections do not change the original conclusions of the article. The online article has been corrected on October 9, 2026. The errors remain in the PDF and in print.

**Figure:**
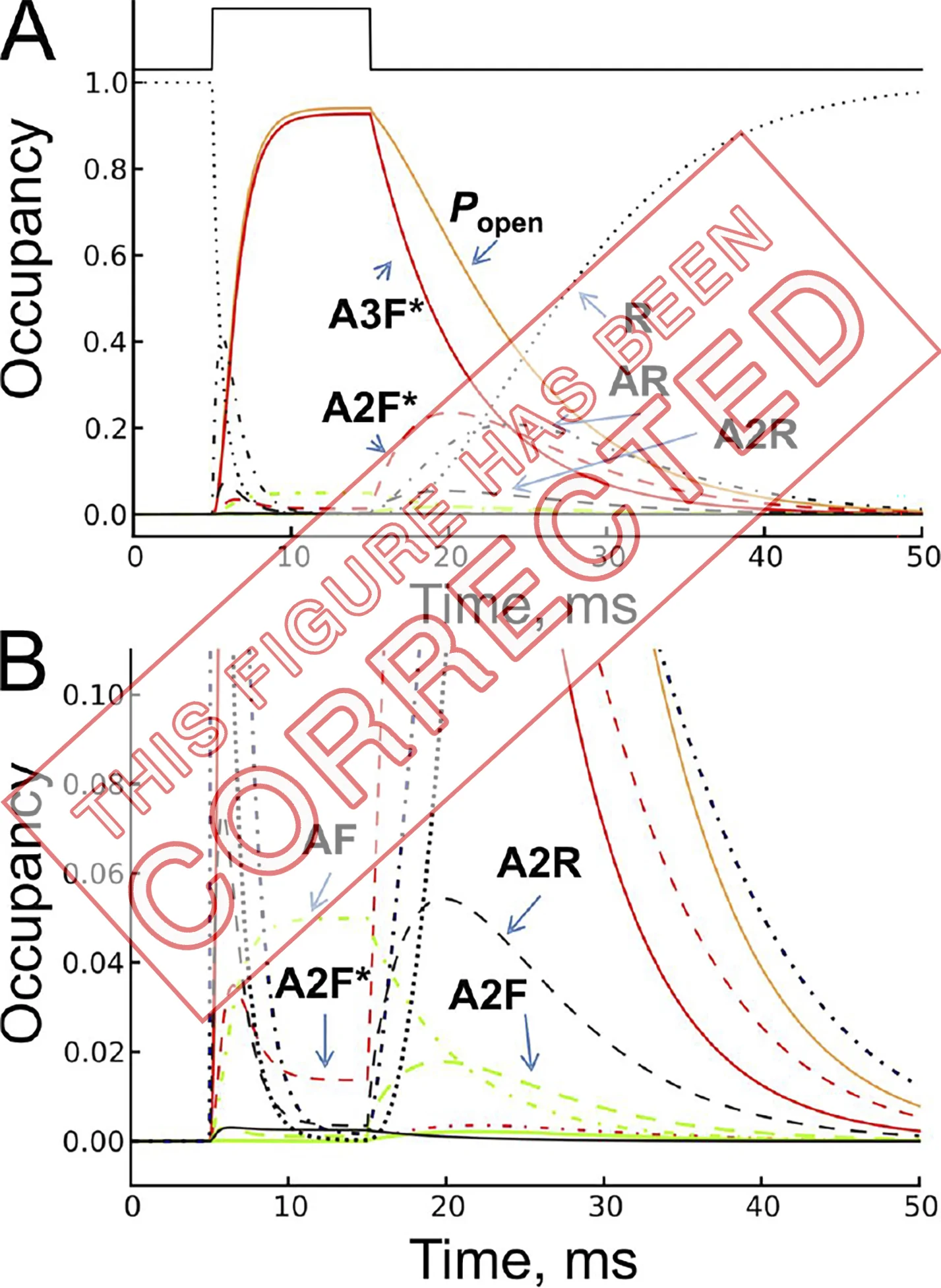
**The time course of occupancies.** The time course of glycine receptor state occupancies is shown over the full range, 0–1, in A, and as an enlarged view of occupancies below 0.1 in B. The occupancies were calculated from Eq. 1 and plotted for all 10 states in the flip mechanism (Fig. 3) after a step in glycine concentration from 0 to 1 mM, and, 10 ms later, a step from 1 mM to zero (the concentration profile is shown on top of panel A as a black solid line). Occupancies of the resting conformation are plotted in black, the flipped conformation is plotted in green, and the open conformation is plotted in red. Solid lines represent triliganded states, dashed lines those of diliganded states, and dashed-dot lines show monoliganded states. Occupancy of the unliganded resting state R (state 10) is plotted as a dotted black line and the total open probability as a solid orange line.

**Figure 6.**
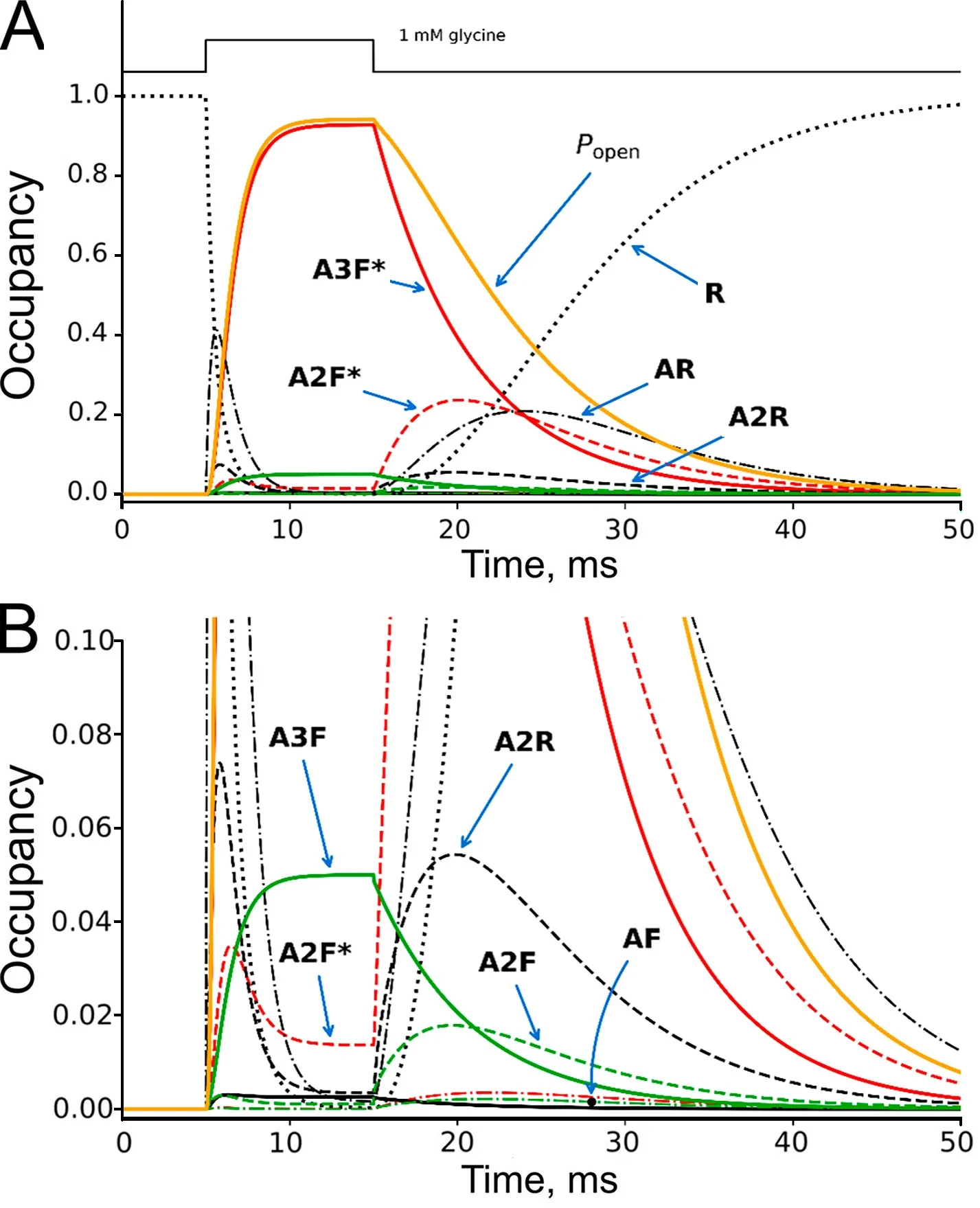
**The time course of occupancies.** The time course of glycine receptor state occupancies is shown over the full range, 0–1, in A, and as an enlarged view of occupancies below 0.1 in B. The occupancies were calculated from Eq. 1 and plotted for all 10 states of the flip mechanism (Fig. 3) after a step in glycine concentration from 0 to 1 mM, and, 10 ms later, a step from 1 mM to zero (the concentration profile is shown on top of panel A as a black solid line).The predicted occupancies were calculated, as in Figs. 7 and 8, using the rate constants from one of the three sets of observations fromBurzomato et al. (2004; the set that was used for the illustrations in that paper), not the averaged values shown in Fig. 3. Occupancies of the resting conformation are plotted in black, the flipped conformation is plotted in green, and the open conformation is plotted in red. Solid lines represent triliganded states, dashed lines those of diliganded states, and dashed-dot lines show monoliganded states. Occupancy of the unliganded resting state R (state 10) is plotted as a dotted black line and the total open probability as a solid orange line.

**Table 1.** Equilibrium occupancies of each state at equilibrium (as a percentage) for two concentrations of glycine

State	1AF*	2A_2_F*	3A_3_F*	4A_3_F	5A_2_F	6AF	7A_3_R	8A_2_R	9AR	10R
10 µM	0.047	0.559	0.381	0.0205	0.042	0.0288	0.001	0.139	6.21	92.57
1 mM	**0.0012**	1.36	92.77	4.99	0.103	0.0007	0.253	0.339	0.151	0.023

The occupancies were calculated, as in Figs. 6 , 7, and 8, using the rate constants from one of the three sets of observations from Burzomato et al. (2004 ; the set that was used for the illustrations in that paper), not the averaged values shown in Fig. 3.

Here are provided links to the code that was used, together with a Jupyter notebook that recomputes every number and calculated figure in the original article (Figs. 6, 7, and 8, Table 1, and the quantities quoted in the text). The notebook is available at https://github.com/DCPROGS/ColquhounLape2012-reproduction (archived at https://doi.org/10.5281/zenodo.21916703). The calculations use SCALCS, pinned to the release https://github.com/DCPROGS/SCALCS/releases/tag/v0.5.1-cl2012. The notebook was drafted with the assistance of a large language model, Anthropic’s Claude Opus 5, running under Claude Code, a command-line tool that lets the model read files, run code, and inspect the results. Essentially all of the notebook code was generated by the model working from short instructions from the authors, which has since been checked and edited throughout. Every decision about what to compute and every acceptance of a result was made by the authors. The model was productive and, on the whole, careful. However, it was also confidently wrong often enough that nothing it asserted could be accepted without being checked. The executed notebook, rendered to a PDF file, accompanies this correction online (Data S1).

## Fig. 6 B

The AF and A_3_F traces in Fig. 6 B had been mistakenly interchanged, and the labels and lines have been corrected. AF and A_3_F are the symbols used for the intermediate “flip” conformation of the receptor with 1 or 3 agonist molecules bound, respectively; see the reaction mechanism in Fig. 3 of Colquhoun and Lape (2012). The line styles followed the labels rather than the traces, so the interchange broke the convention stated in the legend, under which solid lines denote triliganded states and dash-dot lines denote monoliganded states. Thus, the line for A_3_F has been changed from dotted to solid.

At 1 mM glycine, 12 ms into the pulse, the occupancy of A_3_F is 0.0498, and that of AF is 0.0000077, a ratio of ∼6,400. Thus, the plateau at ∼0.05 during the pulse can only be A_3_F. Table 1 of the article gives the corresponding equilibrium occupancies at 1 mM as 4.99% for A_3_F and 0.0007% for AF, which is correct as published.

A corrected Fig. 6 and legend are provided here with the changes indicated. See section 7 in the notebook for the Fig. 6 reproduction code.

The calculations used a single dataset. Fig. 6 and Table 1, like Figs. 7 and 8, were computed from one of the three sets of rate constants of Burzomato et al. (2004) named bgtv2a and are tabulated in section 3 of the notebook, which is the same set that was used for the illustrations in that paper, and not from the averaged rate constants displayed in Fig. 3. Each set of rate constants was obtained by a separate fit of single ion channel recordings using HJCFIT (Colquhoun et al., 2003). The legend to Fig. 7 states this, but Table 1 and the Fig. 6 legend do not, and are amended here.

## Table 1

Also, in Table 1 of this paper, the equilibrium occupancy of 1AF* at 1 mM has been corrected to 0.0012%; the other 19 entries in the table are correct as published. Equilibrium occupancy values are recalculated in section 5 of the notebook. The corrected value appears here in bold. The title to Table 1 has changed, and a legend has been added.

## Text section “A numerical example”

In the text, the probability of the clockwise route at 10 µM is given as 2.8 × 10^−5^ in the “A numerical example” section, whereas the expression that yields the other two probabilities quoted in the same sentence and that reproduces the 1 mM value exactly gives 2.385 × 10^−5^, or 2.4 × 10^−5^ when rounded to two significant figures. The statement in the following sentence, that the other two routes are about 30 times more likely than the clockwise route, holds for 2.4 × 10^−5^ (ratios 30.7 and 32.1) but not for 2.8 × 10^−5^ (26.1 and 27.5). The route probabilities are recalculated in section 11 of the notebook. The affected paragraph is shown below.

“At a low concentration of glycine (10 µM), these routes give probabilities of 7.3 × 10^−4^, 2.8 × 10^−5^2.4 × 10^−5^, and 7.7 × 10^−4^, respectively, so the anticlockwise route and the route via states 8 and 5 are about equally likely, and both are about 30 times more likely than the clockwise route via state 7. The most likely routes at low concentration involve flipping while monoliganded (anticlockwise route) or while diliganded.”

## Supplementary Material

Data S1 is the executed Jupyter notebook containing the data underlying the corrections to Fig. 6, Table 1, and the text.
