# Supplementary material for: Correction: Allosteric coupling in ligand-gated ion channels
Source: J Gen Physiol. 2026 Oct 12;158(6):e20121084408242026c. doi: 10.1085/jgp.20121084408242026c (PMC13656842; doi:10.1085/jgp.20121084408242026c)
Supplement: Supplementary file 1 [file jgp_20121084408242026c_datas1.pdf]

ColquhounLape2012_reproduction

October 6, 2026

Colquhoun & Lape (2012)- full numerical reproduction

Colquhoun, D., and R. Lape. 2012. Allosteric coupling in ligand-gated ion channels. *J. Gen. Physiol.* 140:599–612. doi:10.1085/jgp.201210844

This notebook reproduces every number and figure in the quantitative part of the paper using the SCALCS package:

Item	Content
Rate constants	all 22 rates of the flip mechanism, with units
Q-matrices	at 10 µM and 1 mM glycine
**Table 1**	equilibrium occupancies of the 10 states
**Figure 6**	occupancy time course during a 0 → 1 mM → 0 concentration jump
**Figure 7**	transition probabilities π_{ij}
**Figure 8**	transition frequencies f_{ij} at equilibrium
Text numbers	state lifetimes, binding frequencies, route probabilities, $E/F/K$

Note on the use of AI. The notebook was drafted with the assistance of a large language model, Anthropic's Claude Opus 5, running under Claude Code, a command-line tool that lets the model read files, run code and inspect the results. Essentially all of the notebook code was generated by the model working from short instructions from the authors but has since been checked and edited throughout, and every decision about what to compute, and every acceptance of a result, was made by the authors.

---

1 Which rate constants did the paper use?

Two different sets appear in the paper, and this matters for every number below.

Fig. 3 displays the **mean of fits to three datasets** (Table 5 of Burzomato et al. 2004). This set of rate constants is in `scalcs.samples.samples.GlyR_flip`.

The paper's whole quantitative section- **Table 1 and Figs. 6, 7 and 8**, and every number quoted in the text- was computed from one of the three sets of rate constants of Burzomato et al. (2004), the same set that was used for the illustrations in that paper. Only the legends of **Figs. 7 and 8**

say so. That **Table 1 and Fig. 6** come from the same single dataset is not stated anywhere in the paper.

The reason for using a single set of rate constants is that the averaged rate constants do **not** satisfy microscopic reversibility exactly, because averaging rate constants does not preserve the cycle conditions.

The single dataset named **bgtv2a** is embedded directly below, so this notebook needs no external data file.

1.1 A note on state numbering

Note that the state order is different between paper and SCALCS. However, everything is indexed by state *name*, so the ordering never has to be tracked by hand.

	order
Paper (Table 1, Figs 7–8)	`1 AF*, 2 A2F*, 3 A3F*, 4 A3F, 5 A2F, 6 AF, 7 A3R, 8 A2R, 9 AR, 10 R`
SCALCS	`AF*, A2F*, A3F*, AF, A2F, A3F, A3R, A2R, AR, R`- **states 4 and 6 swapped**

2 Setup

[1]:
```
import os

import numpy as np
import pandas as pd
import matplotlib.pyplot as plt

from scalcs import qmatlib as qml
from scalcs import cjumps, popen

# The tables below are rendered through the pandas Styler (df.style.format),
# which needs jinja2. jinja2 arrives as a dependency of Jupyter itself in most
# installs, but not with a bare `pip install scalcs pandas`, so check for it
# here rather than failing on the first table many cells further down.
try:
    import jinja2
except ImportError:
    raise ImportError(
        "This notebook renders its tables with the pandas Styler, which "
        "requires jinja2.  Install it with `pip install jinja2` (or use the "
        "environment.yml shipped alongside this notebook) and re-run."
    )

from IPython.display import display
```

```python
# Show a styled table.
#
# A Styler offers only text/html, so exporting the notebook to LaTeX (and hence
# to PDF) drops every table and prints the repr of the Styler object instead.
# Emitting text/latex alongside the HTML fixes that: the browser takes the HTML
# and nbconvert's LaTeX exporter takes the LaTeX.
#
# to_latex() does not escape, and these tables carry characters that are special
# outside maths mode - "%" in headings like "bgtv2a % err", and "^" in units
# like "s^-1" - so escaping has to be asked for. It is applied to the index and
# the column headings, and to the text columns; the numeric columns keep the
# formats set on them. Cell shading is not carried into LaTeX, which affects
# only the one table that uses a colour scale.
def show(styler):
    bundle = {'text/html': styler.to_html()}
    try:
        # Cell shading is dropped for LaTeX. Left in, it emits either raw CSS
        # (\background-color#ffebe, not LaTeX at all) or \cellcolor, which
        # needs xcolor's table option that the nbconvert template does not
        # load. Clear the style functions in place - replacing ctx with a plain
        # dict instead breaks to_latex, which expects a defaultdict.
        styler._todo = []
        styler.ctx.clear()

        esc = styler.format_index(escape='latex', axis=0) \
                    .format_index(escape='latex', axis=1)
        # Test for "not numeric" rather than "dtype is object": pandas 3 gives
        # string columns the new str dtype, so an object test finds nothing and
        # the units column ships s^-1 unescaped.
        text_cols = [c for c in styler.data.columns
                     if not pd.api.types.is_numeric_dtype(styler.data[c])]
        if text_cols:
            esc = esc.format(escape='latex', subset=text_cols)
        tex = esc.to_latex(hrules=True)
        # The Q matrices are eleven columns wide and run off the page. Shrink a
        # table to the text width only if it is wider; narrow ones are left at
        # their natural size.
        tex = ('\\resizebox{\\ifdim\\width>\\linewidth\\linewidth'
               '\\else\\width\\fi}{!}{%%\n%s}\n' % tex.strip())
        bundle['text/latex'] = tex
    except Exception:
        pass
    display(bundle, raw=True)

pd.set_option('display.width', 200)
```

```
pd.set_option('display.max_columns', 40)
plt.rcParams.update({'figure.dpi': 110, 'font.size': 10})

C_LOW, C_HIGH = 10e-6, 1e-3          # the two concentrations used in the paper

# The paper's ordering (Table 1, Figs 7-8)
PAPER_ORDER = ['AF*', 'A2F*', 'A3F*', 'A3F', 'A2F', 'AF',
               'A3R', 'A2R', 'AR', 'R']
PNO = {n: i + 1 for i, n in enumerate(PAPER_ORDER)}


# Equilibrium occupancies p(inf), from p.Q = 0 with sum(p) = 1.
#
# Uses the reduced-Q solve. Some SCALCS releases default pinf() to the
# extended-Q method, which appends a column of ones and inverts S.S^T -
# squaring the condition number, and leaving sum(p) - 1 near 3e-9 instead of
# exactly 0. Both methods ship in those releases, so preferring the reduced-Q
# one makes this notebook give identical numbers on every version.
def equilibrium_p(Q):
    fn = getattr(qml, 'pinf_reduceQ', None) or qml.pinf
    return fn(Q)
```

2.1 The rate constants of the bgtv2a dataset

The mechanism is defined here in full, so this notebook is self-contained and needs no external data file. The rate constants are those of the single Burzomato et al. (2004) dataset **bgtv2a**.

They are transcribed from the DC-format file `bgtv2a.mec`, which stores them as 32-bit floats, hence the long decimal expansions, which are exact rather than spuriously precise. Six significant figures would be ample; the full expansions are kept only so that anyone re-running this notebook gets bit-identical numbers.

The paper's flip notation (`AF/A2F/A3F`, `AF*/A2F*/A3F*`) is used throughout.

[2]:
```
from scalcs import mechanism


def GlyR_flip_bgtv2a():
    """The single Burzomato et al. (2004) dataset "bgtv2a".

    These are the rate constants from which Colquhoun & Lape (2012) computed
    Table 1 and Figures 6, 7 and 8, and every number quoted in the text.
    Figure 3 of that paper shows something different: the mean of fits to
    three datasets (that average is `scalcs.samples.samples.GlyR_flip`).
    """
    mectitle = 'flip, Burzomato2004'
    ratetitle = 'bgtv2a - the single dataset used in Colquhoun & Lape (2012)'
```

```
AFS   = mechanism.State('A', 'AF*', 4e-11)
A2FS  = mechanism.State('A', 'A2F*', 4e-11)
A3FS  = mechanism.State('A', 'A3F*', 4e-11)
AF    = mechanism.State('B', 'AF', 0)
A2F   = mechanism.State('B', 'A2F', 0)
A3F   = mechanism.State('B', 'A3F', 0)
A3R   = mechanism.State('B', 'A3R', 0)
A2R   = mechanism.State('B', 'A2R', 0)
AR    = mechanism.State('B', 'AR', 0)
R     = mechanism.State('C', 'R', 0)

RateList = [
    # opening / shutting
    mechanism.Rate(3690.69189453125, AFS, AF, name='alpha1',
                   limits=[1e-15, 1e+7]),
    mechanism.Rate(6059.1357421875, AF, AFS, name='beta1',
                   limits=[1e-15, 1e+7]),
    mechanism.Rate(2472.840576171875, A2FS, A2F, name='alpha2',
                   limits=[1e-15, 1e+7]),
    mechanism.Rate(32815.8515625, A2F, A2FS, name='beta2',
                   limits=[1e-15, 1e+7]),
    mechanism.Rate(6975.74609375, A3FS, A3F, name='alpha3',
                   limits=[1e-15, 1e+7]),
    mechanism.Rate(129611.671875, A3F, A3FS, name='beta3',
                   limits=[1e-15, 1e+7]),
    # flipping (gamma = flipped->resting, delta = resting->flipped)
    mechanism.Rate(1064.94287109375, A3F, A3R, name='gamma3',
                   limits=[1e-15, 1e+7]),
    mechanism.Rate(21028.595703125, A3R, A3F, name='delta3',
                   limits=[1e-15, 1e+7]),
    mechanism.Rate(20826.802734375, A2F, A2R, name='gamma2',
                   limits=[1e-15, 1e+7]),
    mechanism.Rate(6304.60302734375, A2R, A2F, name='delta2',
                   limits=[1e-15, 1e+7]),
    mechanism.Rate(31142.638671875, AF, AR, name='gamma1',
                   limits=[1e-15, 1e+7]),
    mechanism.Rate(144.52456665039062, AR, AF, name='delta1',
                   limits=[1e-15, 1e+7]),
    # binding to the flipped conformation
    mechanism.Rate(3417.26611328125, A3F, A2F, name='3kf(-3)',
                   limits=[1e-15, 1e+7]),
    mechanism.Rate(166282256.0, A2F, A3F, name='kf(+3)', eff='c',
                   limits=[1e-15, 1e+10]),
    mechanism.Rate(2278.177490234375, A2F, AF, name='2kf(-2)',
                   limits=[1e-15, 1e+7]),
    mechanism.Rate(332564512.0, AF, A2F, name='2kf(+2)', eff='c',
                   limits=[1e-15, 1e+10]),
```

```
        # binding to the resting conformation
        mechanism.Rate(826.9252319335938, A3R, A2R, name='3k(-3)',
                       limits=[1e-15, 1e+7]),
        mechanism.Rate(616857.0625, A2R, A3R, name='k(+3)', eff='c',
                       limits=[1e-15, 1e+10]),
        mechanism.Rate(551.2835083007812, A2R, AR, name='2k(-2)',
                       limits=[1e-15, 1e+7]),
        mechanism.Rate(1233714.125, AR, A2R, name='2k(+2)', eff='c',
                       limits=[1e-15, 1e+10]),
        mechanism.Rate(275.6417541503906, AR, R, name='k(-1)',
                       limits=[1e-15, 1e+7]),
        mechanism.Rate(1850571.125, R, AR, name='3k(+1)', eff='c',
                       limits=[1e-15, 1e+10]),
        ]

    CycleList = [mechanism.Cycle(['A2F', 'AF', 'AR', 'A2R']),
                 mechanism.Cycle(['A3F', 'A2F', 'A2R', 'A3R'])]

    return mechanism.Mechanism(RateList, CycleList,
                              mtitle=mectitle, rtitle=ratetitle)


def GlyR_flip_fig3():
    """The Fig. 3 rate constants: means of fits to three datasets.

    Same values as scalcs.samples.samples.GlyR_flip, embedded here so the
    notebook does not depend on where a given SCALCS release puts its
    samples package (scalcs.samples vs top-level samples).

    These are what Fig. 3 of the paper displays. They are NOT what Table 1
    and Figs 6-8 were computed from; the summary at the end of the notebook
    shows how far out they are.
    """
    mectitle = '3 site flip'
    ratetitle = 'Burzomato 2004 heteromeric GlyR, Fig 3 averages'

    AFS   = mechanism.State('A', 'AF*', 6e-11)
    A2FS  = mechanism.State('A', 'A2F*', 6e-11)
    A3FS  = mechanism.State('A', 'A3F*', 6e-11)
    AF    = mechanism.State('B', 'AF', 0.0)
    A2F   = mechanism.State('B', 'A2F', 0.0)
    A3F   = mechanism.State('B', 'A3F', 0.0)
    A3R   = mechanism.State('B', 'A3R', 0.0)
    A2R   = mechanism.State('B', 'A2R', 0.0)
    AR    = mechanism.State('B', 'AR', 0.0)
    R     = mechanism.State('C', 'R', 0.0)
```

```python
RateList = [
    mechanism.Rate(3400.0, AFS, AF, name='alpha1',
                   limits=[1e-15, 1e+7]),
    mechanism.Rate(4200.0, AF, AFS, name='beta1',
                   limits=[1e-15, 1e+7]),
    mechanism.Rate(2100.0, A2FS, A2F, name='alpha2',
                   limits=[1e-15, 1e+7]),
    mechanism.Rate(28000.0, A2F, A2FS, name='beta2',
                   limits=[1e-15, 1e+7]),
    mechanism.Rate(7000.0, A3FS, A3F, name='alpha3',
                   limits=[1e-15, 1e+7]),
    mechanism.Rate(129000.0, A3F, A3FS, name='beta3',
                   limits=[1e-15, 1e+7]),
    mechanism.Rate(900.0, A3F, A3R, name='gamma3',
                   limits=[1e-15, 1e+7]),
    mechanism.Rate(20900.0, A3R, A3F, name='delta3',
                   limits=[1e-15, 1e+7]),
    mechanism.Rate(3600.0, A3F, A2F, name='3kf(-3)',
                   limits=[1e-15, 1e+7]),
    mechanism.Rate(150000000.0, A2F, A3F, name='kf(+3)', eff='c',
                   limits=[1e-15, 1e+10]),
    mechanism.Rate(18000.0, A2F, A2R, name='gamma2',
                   limits=[1e-15, 1e+7]),
    mechanism.Rate(6800.0, A2R, A2F, name='delta2',
                   limits=[1e-15, 1e+7]),
    mechanism.Rate(2400.0, A2F, AF, name='2kf(-2)',
                   limits=[1e-15, 1e+7]),
    mechanism.Rate(300000000.0, AF, A2F, name='2kf(+2)', eff='c',
                   limits=[1e-15, 1e+10]),
    mechanism.Rate(29000.0, AF, AR, name='gamma1',
                   limits=[1e-15, 1e+7]),
    mechanism.Rate(180.0, AR, AF, name='delta1',
                   limits=[1e-15, 1e+7]),
    mechanism.Rate(900.0, A3R, A2R, name='3k(-3)',
                   limits=[1e-15, 1e+7]),
    mechanism.Rate(590000.0, A2R, A3R, name='k(+3)', eff='c',
                   limits=[1e-15, 1e+10]),
    mechanism.Rate(600.0, A2R, AR, name='2k(-2)',
                   limits=[1e-15, 1e+7]),
    mechanism.Rate(1180000.0, AR, A2R, name='2k(+2)', eff='c',
                   limits=[1e-15, 1e+10]),
    mechanism.Rate(300.0, AR, R, name='k(-1)',
                   limits=[1e-15, 1e+7]),
    mechanism.Rate(1770000.0, R, AR, name='3k(+1)', eff='c',
                   limits=[1e-15, 1e+10]),
    ]
```

```
    CycleList = [mechanism.Cycle(['A2F', 'AF', 'AR', 'A2R']),
                 mechanism.Cycle(['A3F', 'A2F', 'A2R', 'A3R'])]

    return mechanism.Mechanism(RateList, CycleList,
                               mtitle=mectitle, rtitle=ratetitle)
```

[3]:

```
class Model:
    # Thin wrapper: a Mechanism plus a name->index map in the paper's notation.

    def __init__(self, mec, rename=None, label=''):
        self.mec = mec
        self.label = label
        rename = rename or {}
        self.names = [rename.get(s.name, s.name) for s in mec.States]
        self.idx = {n: i for i, n in enumerate(self.names)}
        self.perm = [self.idx[n] for n in PAPER_ORDER]

    def Q(self, c):
        self.mec.set_eff('c', c)
        return self.mec.Q.copy()

    def pinf(self, c):
        return equilibrium_p(self.Q(c))

    def i(self, name):
        return self.idx[name]

    # rate constant looked up by the (from, to) state pair
    def rate_by_edge(self):
        out = {}
        rename = {s.name: n for s, n in zip(self.mec.States, self.names)}
        for r in self.mec.Rates:
            out[(rename[r.State1.name], rename[r.State2.name])] = r
        return out


BG = Model(GlyR_flip_bgtv2a(), None, 'bgtv2a (single dataset)')
AV = Model(GlyR_flip_fig3(), None, 'Fig 3 averages')

print('primary   : %s / %s' % (BG.mec.mtitle.strip(), BG.mec.rtitle.strip()))
print('comparison: %s / %s' % (AV.mec.mtitle.strip(), AV.mec.rtitle.strip()))
print()
print('states (paper notation, SCALCS order):', BG.names)
print('k = %d, kA = %d open states, %d rate constants'
      % (BG.mec.k, BG.mec.kA, len(BG.mec.Rates)))
print('identical topology to GlyR_flip:',
```

```
    {(a, b) for a, b in BG.rate_by_edge()} == {(a, b) for a, b in AV.
↪rate_by_edge()})
```

```
primary   : flip, Burzomato2004 / bgtv2a - the single dataset used in Colquhoun
& Lape (2012)
comparison: 3 site flip / Burzomato 2004 heteromeric GlyR, Fig 3 averages

states (paper notation, SCALCS order): ['AF*', 'A2F*', 'A3F*', 'AF', 'A2F',
'A3F', 'A3R', 'A2R', 'AR', 'R']
k = 10, kA = 3 open states, 22 rate constants
identical topology to GlyR_flip: True
```

2.2 Two equations from the paper, and a version shim

π_{ij} and f_{ij} are implemented here directly from **Eqs. 2 and 3** of the paper rather than called from a library, so the arithmetic behind **Figures 7 and 8** is visible in the notebook and the notebook does not depend on which SCALCS version supplies them.

The concentration-jump helper adapts to whichever API the installed SCALCS offers. The pinned release v0.5.1-cl2012 provides the procedural `solve_jump`/`pulse_square`; later releases provide `solve`/`SquarePulse`. Both describe the same pulse and integrate it with the same solver and tolerances, and they agree to better than one part in 10^6 on every quantity reported here, so nothing printed in this notebook depends on which one is used. The residual difference comes from the equilibrium occupancies that seed the integration, which differ in their last few digits between versions, not from the pulse description.

[4]:
```
NEW_JUMP_API = hasattr(cjumps, 'SquarePulse')


def transition_probability(Q):
    """Eq. 2: the probability that the next transition out of state i is to j.

        pi_ij = q_ij / sum_{j != i} q_ij = -q_ij / q_ii

    Rows sum to 1. Independent of time; no assumption of equilibrium.
    """
    Q = np.asarray(Q, dtype=float)
    pi = Q / -np.diag(Q)[:, None]
    np.fill_diagonal(pi, 0.0)
    return pi


def transition_frequency(Q, p):
    """Eq. 3: the number of i -> j transitions per second, f_ij = p_i * q_ij.

    Evaluated with the equilibrium occupancies p(inf) for Figure 8.
    """
    Q = np.asarray(Q, dtype=float)
```

```
    f = np.asarray(p, dtype=float)[:, None] * Q
    np.fill_diagonal(f, 0.0)
    return f


def square_pulse_jump(mec, cmax, width, prepulse, reclen, step, cb=0.0):
    """Response to a rectangular concentration pulse -> (t, c, Popen, P)."""
    if NEW_JUMP_API:
        pulse = cjumps.SquarePulse(cmax=cmax, width=width,
                                   prepulse=prepulse, cb=cb)
        r = cjumps.solve(mec, pulse, reclen=reclen, step=step, method='ode')
        return r.t, r.c, r.Popen, r.P
    cargs = (cmax, cb, prepulse, width)
    return cjumps.solve_jump(mec, reclen, step, cjumps.pulse_square, cargs)


print('jump API in this SCALCS : %s'
      % ('solve / SquarePulse' if NEW_JUMP_API else 'solve_jump /␣
 ↪pulse_square'))
```

```
jump API in this SCALCS : solve_jump / pulse_square
```

3 Rate constants

The 22 rate constants of the flip mechanism (Fig. 3 of the paper). Association steps are concentration dependent and carry units of M^{-1} s^{-1}; the values already include the statistical factor for the number of vacant (or occupied) binding sites, e.g. `3k(+1)` $= 3 \times k_{+1}$.

Rates are matched between the two mechanisms **by their (from, to) state pair**, which is unambiguous, rather than by name.

[5]:
```
bg_edges, av_edges = BG.rate_by_edge(), AV.rate_by_edge()

rows = []
for (a, b), r_av in av_edges.items():
    r_bg = bg_edges[(a, b)]
    conc_dep = r_av._effectors[0] is not None
    rows.append({
        'name': r_av.name.strip(),
        'from': a, 'to': b,
        'bgtv2a': r_bg.unit_rate(),
        'Fig 3 averaged': r_av.unit_rate(),
        'ratio': r_bg.unit_rate() / r_av.unit_rate(),
        'units': 'M^-1 s^-1' if conc_dep else 's^-1',
    })
rates = pd.DataFrame(rows)
show(rates.style.format({'bgtv2a': '{:.7g}', 'Fig 3 averaged': '{:.6g}',␣
 ↪'ratio': '{:.3f}'}))
```

	name	from	to	bgtv2a	Fig 3 averaged	ratio	units
0	alpha1	AF*	AF	3690.692	3400	1.085	s^-1
1	beta1	AF	AF*	6059.136	4200	1.443	s^-1
2	alpha2	A2F*	A2F	2472.841	2100	1.178	s^-1
3	beta2	A2F	A2F*	32815.85	28000	1.172	s^-1
4	alpha3	A3F*	A3F	6975.746	7000	0.997	s^-1
5	beta3	A3F	A3F*	129611.7	129000	1.005	s^-1
6	gamma3	A3F	A3R	1064.943	900	1.183	s^-1
7	delta3	A3R	A3F	21028.6	20900	1.006	s^-1
8	3kf(-3)	A3F	A2F	3417.266	3600	0.949	s^-1
9	kf(+3)	A2F	A3F	1.662823e+08	1.5e+08	1.109	M^-1 s^-1
10	gamma2	A2F	A2R	20826.8	18000	1.157	s^-1
11	delta2	A2R	A2F	6304.603	6800	0.927	s^-1
12	2kf(-2)	A2F	AF	2278.177	2400	0.949	s^-1
13	2kf(+2)	AF	A2F	3.325645e+08	3e+08	1.109	M^-1 s^-1
14	gamma1	AF	AR	31142.64	29000	1.074	s^-1
15	delta1	AR	AF	144.5246	180	0.803	s^-1
16	3k(-3)	A3R	A2R	826.9252	900	0.919	s^-1
17	k(+3)	A2R	A3R	616857.1	590000	1.046	M^-1 s^-1
18	2k(-2)	A2R	AR	551.2835	600	0.919	s^-1
19	2k(+2)	AR	A2R	1233714	1.18e+06	1.046	M^-1 s^-1
20	k(-1)	AR	R	275.6418	300	0.919	s^-1
21	3k(+1)	R	AR	1850571	1.77e+06	1.046	M^-1 s^-1

3.1 Derived equilibrium constants

These are the quantities marked in brown on Fig. 3 and quoted in the text.

Fig. 3's E, F and K values are *means over the three datasets*, and the paper's Fig. 2 legend states they were obtained by averaging the equilibrium constants separately rather than as ratios of the averaged rate constants. So `bgtv2a`, being one of the three sets that were averaged, legitimately differs from their mean.

[6]:
```
def eq_constants(m):
    e = m.rate_by_edge()
    q = lambda a, b: e[(a, b)].unit_rate()
    E1, E2, E3 = q('AF','AF*')/q('AF*','AF'), q('A2F','A2F*')/q('A2F*','A2F'),␣
↪q('A3F','A3F*')/q('A3F*','A3F')
    F1, F2, F3 = q('AR','AF')/q('AF','AR'), q('A2R','A2F')/q('A2F','A2R'),␣
↪q('A3R','A3F')/q('A3F','A3R')
    KR = q('AR','R') / (q('R','AR')/3)
    KF = (q('A3F','A2F')/3) / q('A2F','A3F')
    return {'E1': E1, 'E2': E2, 'E3': E3, 'E2/E1': E2/E1, 'E3/E2': E3/E2,
            'F1': F1, 'F2': F2, 'F3': F3, 'K_R (uM)': KR*1e6, 'K_F (uM)':␣
↪KF*1e6}


eq = pd.DataFrame({
```

```
    'published (Fig 3)': {'E1': 1.3, 'E2': 13, 'E3': 20, 'E2/E1': 10, 'E3/E2':␣
↪1.5,
                          'F1': 0.006, 'F2': 0.4, 'F3': 27, 'K_R (uM)': 520,␣
↪'K_F (uM)': 8},
    'bgtv2a': eq_constants(BG),
})
E1, E2, E3 = (eq_constants(BG)[k] for k in ('E1', 'E2', 'E3'))
F1, F2, F3 = (eq_constants(BG)[k] for k in ('F1', 'F2', 'F3'))
KR, KF = eq_constants(BG)['K_R (uM)']/1e6, eq_constants(BG)['K_F (uM)']/1e6
show(eq.style.format('{:.4g}'))
```

	published (Fig 3)	bgtv2a
E1	1.3	1.642
E2	13	13.27
E3	20	18.58
E2/E1	10	8.083
E3/E2	1.5	1.4
F1	0.006	0.004641
F2	0.4	0.3027
F3	27	19.75
K_R (uM)	520	446.8
K_F (uM)	8	6.85

The text uses F_3 to quantify the error made by lumping `A3R` and `A3F` into a single shut state: the apparent dissociation rate constant is the true one multiplied by $1/(1+F_3)$, and the effective opening rate constant differs by $F_3/(1+F_3)$.

[7]:
```
print('F3           = %.4f' % F3)
print('1/(1 + F3)   = %.4f   <- fraction of [A3R, A3F] that is A3R' % (1/(1+F3)))
print('F3/(1 + F3) = %.4f' % (F3/(1+F3)))
print()
print('E2/E1 = %.3g and E3/E2 = %.3g  -> "far from constant", so either the␣
↪model'
      % (E2/E1, E3/E2))
print('is wrong or the opening reaction does not obey microscopic reversibility.
↪')
```

```
F3           = 19.7462
1/(1 + F3)   = 0.0482   <- fraction of [A3R, A3F] that is A3R
F3/(1 + F3) = 0.9518

E2/E1 = 8.08 and E3/E2 = 1.4  -> "far from constant", so either the model
is wrong or the opening reaction does not obey microscopic reversibility.
```

4 The Q-matrices

Q holds the transition rate constants: the off-diagonal element q_{ij} is the rate constant for $i \to j$ (s^{-1}), and the diagonal is defined so every row sums to zero,

$$q_{ii} = -\sum_{j \neq i} q_{ij}.$$

Shown in the paper's state order, so row/column 1 is `AF*` and row/column 10 is `R`.

[8]:
```
def Q_frame(m, c):
    Q = m.Q(c)[np.ix_(m.perm, m.perm)]
    lab = ['%d %s' % (PNO[n], n) for n in PAPER_ORDER]
    return pd.DataFrame(Q, index=lab, columns=lab)

for c, tag in ((C_LOW, '10 uM'), (C_HIGH, '1 mM')):
    print('Q at %-6s : max |row sum| = %.2e   (must be 0)'
          % (tag, np.abs(BG.Q(c).sum(axis=1)).max()))
```

```
Q at 10 uM  : max |row sum| = 0.00e+00   (must be 0)
Q at 1 mM   : max |row sum| = 0.00e+00   (must be 0)
```

[9]:
```
print('Q at 10 uM glycine  (s^-1)   [bgtv2a]')
show(Q_frame(BG, C_LOW).style.format('{:.6g}'))
```

```
Q at 10 uM glycine  (s^-1)   [bgtv2a]
```

	1 AF*	2 A2F*	3 A3F*	4 A3F	5 A2F	6 AF	7 A3R	8 A2R	9 AR	10 R
1 AF*	-3690.69	0	0	0	0	3690.69	0	0	0	0
2 A2F*	0	-2472.84	0	0	2472.84	0	0	0	0	0
3 A3F*	0	0	-6975.75	6975.75	0	0	0	0	0	0
4 A3F	0	0	129612	-134094	3417.27	0	1064.94	0	0	0
5 A2F	0	32815.9	0	1662.82	-57583.7	2278.18	0	20826.8	0	0
6 AF	6059.14	0	0	0	3325.65	-40527.4	0	0	31142.6	0
7 A3R	0	0	0	21028.6	0	0	-21855.5	826.925	0	0
8 A2R	0	0	0	0	6304.6	0	6.16857	-6862.06	551.284	0
9 AR	0	0	0	0	0	144.525	0	12.3371	-432.503	275.642
10 R	0	0	0	0	0	0	0	0	18.5057	-18.5057

[10]:
```
print('Q at 1 mM glycine  (s^-1)   [bgtv2a]')
show(Q_frame(BG, C_HIGH).style.format('{:.6g}'))
```

```
Q at 1 mM glycine  (s^-1)   [bgtv2a]
```

	1 AF*	2 A2F*	3 A3F*	4 A3F	5 A2F	6 AF	7 A3R	8 A2R	9 AR	10 R
1 AF*	-3690.69	0	0	0	0	3690.69	0	0	0	0
2 A2F*	0	-2472.84	0	0	2472.84	0	0	0	0	0
3 A3F*	0	0	-6975.75	6975.75	0	0	0	0	0	0
4 A3F	0	0	129612	-134094	3417.27	0	1064.94	0	0	0
5 A2F	0	32815.9	0	166282	-222203	2278.18	0	20826.8	0	0
6 AF	6059.14	0	0	0	332565	-369766	0	0	31142.6	0
7 A3R	0	0	0	21028.6	0	0	-21855.5	826.925	0	0
8 A2R	0	0	0	0	6304.6	0	616.857	-7472.74	551.284	0
9 AR	0	0	0	0	0	144.525	0	1233.71	-1653.88	275.642
10 R	0	0	0	0	0	0	0	0	1850.57	-1850.57

Only the six association steps differ between the two matrices- they scale linearly with concentration (100-fold here). Everything else is identical.

[11]:
```
QL, QH = BG.Q(C_LOW), BG.Q(C_HIGH)
changed = [(BG.names[i], BG.names[j], QL[i, j], QH[i, j], QH[i, j]/QL[i, j])
           for i in range(BG.mec.k) for j in range(BG.mec.k)
           if i != j and not np.isclose(QL[i, j], QH[i, j])]
pd.DataFrame(changed, columns=['from', 'to', 'q at 10 uM', 'q at 1 mM',␣
 ↪'ratio'])
```

[11]:

	from	to	q at 10 uM	q at 1 mM	ratio
0	AF	A2F	3325.645120	332564.512000	100.0
1	A2F	A3F	1662.822560	166282.256000	100.0
2	A2R	A3R	6.168571	616.857062	100.0
3	AR	A2R	12.337141	1233.714125	100.0
4	R	AR	18.505711	1850.571125	100.0

5 Table 1- equilibrium occupancies

At equilibrium the occupancies $p(\infty)$ satisfy $p(\infty)Q = 0$ with $\sum_i p_i = 1$. This is solved by the `equilibrium_p` helper defined in the setup above, which prefers `qmatlib.pinf_reduceQ`, the reduced-Q solve, over the extended-Q method, so that the same digits come out on every SCALCS version.

[12]:
```
PUB_T1 = {'AF*': (0.047, 0.0015), 'A2F*': (0.559, 1.36), 'A3F*': (0.381, 92.77),
          'A3F': (0.0205, 4.99), 'A2F': (0.042, 0.103), 'AF': (0.0288, 0.0007),
          'A3R': (0.001, 0.253), 'A2R': (0.139, 0.339), 'AR': (6.21, 0.151),
          'R': (92.57, 0.023)}

pinf = {'bg': {c: BG.pinf(c) for c in (C_LOW, C_HIGH)},
        'av': {c: AV.pinf(c) for c in (C_LOW, C_HIGH)}}

t1 = pd.DataFrame([{
    'no.': PNO[n], 'state': n,
    '10uM bgtv2a': 100*pinf['bg'][C_LOW][BG.i(n)],
    '10uM Table 1': PUB_T1[n][0],
    '1mM bgtv2a': 100*pinf['bg'][C_HIGH][BG.i(n)],
```

```
    '1mM Table 1': PUB_T1[n][1],
} for n in PAPER_ORDER]).set_index('no.')

print('sum of occupancies: 10 uM = %.10f,  1 mM = %.10f'
      % (pinf['bg'][C_LOW].sum(), pinf['bg'][C_HIGH].sum()))
show(t1.style.format({c: '{:.4g}' for c in t1.columns if c != 'state'}))
```

```
sum of occupancies: 10 uM = 1.0000000000,  1 mM = 1.0000000000
```

no.	state	10uM bgtv2a	10uM Table 1	1mM bgtv2a	1mM Table 1
1	AF*	0.04735	0.047	0.001154	0.0015
2	A2F*	0.5587	0.559	1.362	1.36
3	A3F*	0.3806	0.381	92.77	92.77
4	A3F	0.02049	0.0205	4.993	4.99
5	A2F	0.0421	0.042	0.1026	0.103
6	AF	0.02884	0.0288	0.0007029	0.0007
7	A3R	0.001037	0.001	0.2529	0.253
8	A2R	0.1391	0.139	0.339	0.339
9	AR	6.215	6.21	0.1515	0.151
10	R	92.57	92.57	0.02256	0.023

Nineteen of the twenty entries match to the precision printed in Table 1.

The exception is `AF*` at 1 mM, and it looks like a second misprint rather than a difference in the calculation:

> Table 1 gives **0.0015 %**. `bgtv2a` gives **0.001154 %**, which would print as 0.0012. The Fig. 3 averaged rate constants give 0.001196 %, so neither candidate dataset produces 0.0015. Every other entry in the same column agrees to better than 0.5 %, including `AF` at 0.0007 % and `R` at 0.023 %, so the agreement does not break down for small occupancies in general.

The text's summary of this table: at 1 mM most receptors (93 %) are in the triliganded open state, whereas at 10 µM most (93 %) are still unliganded, and there are *more diliganded than triliganded* open channels.

[13]:
```
for c, tag in ((C_LOW, '10 uM'), (C_HIGH, '1 mM')):
    p = pinf['bg'][c]
    print('%-6s  Popen = %.5f    A2F* = %.4g %%    A3F* = %.4g %%    -> %s'
          % (tag, p[:BG.mec.kA].sum(), 100*p[BG.i('A2F*')], 100*p[BG.i('A3F*')],
             'more diliganded' if p[BG.i('A2F*')] > p[BG.i('A3F*')] else 'more␣
↪triliganded'))
```

```
10 uM   Popen = 0.00987    A2F* = 0.5587 %    A3F* = 0.3806 %    -> more diliganded
1 mM    Popen = 0.94138    A2F* = 1.362 %    A3F* = 92.77 %    -> more triliganded
```

6 Mean lifetimes of the individual states

The mean lifetime of a single sojourn in state i is $-1/q_{ii}$ (Colquhoun & Hawkes 1995b, Eq. 24). States that can only be left by a concentration-independent step have the same lifetime at both concentrations.

The paper quotes the mean lifetime of the resting state R at 1 mM as $1/q_{10,9} = 0.54$ ms.

[14]:
```
lif = pd.DataFrame([{
    'no.': PNO[n], 'state': n,
    '10 uM (ms)': -1e3 / BG.Q(C_LOW)[BG.i(n), BG.i(n)],
    '1 mM (ms)': -1e3 / BG.Q(C_HIGH)[BG.i(n), BG.i(n)],
} for n in PAPER_ORDER]).set_index('no.')
show(lif.style.format({'10 uM (ms)': '{:.4g}', '1 mM (ms)': '{:.4g}'}))
```

no.	state	10 uM (ms)	1 mM (ms)
1	AF*	0.271	0.271
2	A2F*	0.4044	0.4044
3	A3F*	0.1434	0.1434
4	A3F	0.007457	0.007457
5	A2F	0.01737	0.0045
6	AF	0.02467	0.002704
7	A3R	0.04576	0.04576
8	A2R	0.1457	0.1338
9	AR	2.312	0.6046
10	R	54.04	0.5404

[15]:
```
tR = -1e3 / BG.Q(C_HIGH)[BG.i('R'), BG.i('R')]
tR_av = -1e3 / AV.Q(C_HIGH)[AV.i('R'), AV.i('R')]
print('mean lifetime of R at 1 mM')
print('  bgtv2a           %.4f ms   <- paper 0.54 ms' % tR)
print('  averaged rates   %.4f ms' % tR_av)
print()
print('shortest-lived state is A3F at %.1f us - "the occupancy of shut flipped'
      % (-1e6 / BG.Q(C_HIGH)[BG.i('A3F'), BG.i('A3F')]))
print('states is low at all times because of their short mean lifetime".')
```

```
mean lifetime of R at 1 mM
  bgtv2a           0.5404 ms   <- paper 0.54 ms
  averaged rates   0.5650 ms

shortest-lived state is A3F at 7.5 us - "the occupancy of shut flipped
states is low at all times because of their short mean lifetime".
```

7 Figure 6- the time course of occupancies

A rectangular concentration pulse: glycine steps $0 \to 1$ mM at $t = 5$ ms and back to 0 at $t = 15$ ms. All receptors start in the unliganded resting state R. Occupancies follow Eq. 1 of the paper,

$$p(t) = p(0)\exp(Qt).$$

Plotting conventions are those of the published legend: resting states in black, flipped in green, open in red; solid = triliganded, dashed = diliganded, dash-dot = monoliganded; R dotted black and P_{open} solid orange.

Traces are named in the panels rather than in a legend box, as in the published figure, which labels six of them in A and four in B. The labels here are the same, with two differences: what the published panel B labels `AF` is `A3F` (see below), and `AF` itself is labelled at its true position, which is hard against the abscissa.

[16]:
```
CMAX, PREPULSE, WIDTH, RECLEN, STEP = 1e-3, 5e-3, 10e-3, 50e-3, 5e-6

from types import SimpleNamespace

_t, _c, _Popen, _P = square_pulse_jump(BG.mec, CMAX, WIDTH, PREPULSE,
                                       RECLEN, STEP)
jump = SimpleNamespace(t=_t, c=_c, Popen=_Popen, P=_P)
t_ms = jump.t * 1e3

print('occupancies sum to 1 at every time point: min %.8f  max %.8f'
      % (jump.P.sum(axis=0).min(), jump.P.sum(axis=0).max()))
```

```
occupancies sum to 1 at every time point: min 1.00000000  max 1.00000000
```

[17]:
```
STYLE = {   # colour, linestyle, linewidth, label
    'A3F*': ('red',     '-',  1.8, r'$A_3F^*$'),
    'A2F*': ('red',     '--', 1.5, r'$A_2F^*$'),
    'AF*':  ('red',     '-.', 1.2, r'$AF^*$'),
    'A3F':  ('#00b000', '-',  1.8, r'$A_3F$'),
    'A2F':  ('#00b000', '--', 1.5, r'$A_2F$'),
    'AF':   ('#00b000', '-.', 1.2, r'$AF$'),
    'A3R':  ('black',   '-',  1.8, r'$A_3R$'),
    'A2R':  ('black',   '--', 1.5, r'$A_2R$'),
    'AR':   ('black',   '-.', 1.2, r'$AR$'),
    'R':    ('black',   ':',  1.8, r'$R$'),
}
DRAW = ['R', 'A3F*', 'A2F*', 'AF*', 'A3R', 'A2R', 'AR', 'A3F', 'A2F', 'AF']

fig, (axA, axB) = plt.subplots(2, 1, figsize=(7.5, 9))
for ax, ylim, panel in ((axA, (-0.02, 1.22), 'A'), (axB, (-0.002, 0.112), 'B')):
    for n in DRAW:
        col, ls, lw, _ = STYLE[n]
```

```
        ax.plot(t_ms, jump.P[BG.i(n)], color=col, ls=ls, lw=lw)
    ax.plot(t_ms, jump.Popen, color='orange', lw=2.0)
    if panel == 'A':
        ax.plot(t_ms, 1.10 + 0.10 * jump.c / CMAX, color='black', lw=1.2)
        ax.set_yticks([0, .2, .4, .6, .8, 1.0])
    else:
        ax.set_yticks([0, .02, .04, .06, .08, .10])
    ax.set_xlim(0, RECLEN * 1e3); ax.set_ylim(*ylim)
    ax.set_xlabel('Time, ms'); ax.set_ylabel('Occupancy')
    ax.spines[['top', 'right']].set_visible(False)
    ax.text(-0.13, 1.0, panel, transform=ax.transAxes,
            fontsize=20, fontweight='bold', va='top')

# Label the traces in the panels, as the published figure does. The published
# figure carries no legend box; it names six traces in A and four in B with
# bold labels and blue arrows. The same ones are named here, with the one
# correction - what the published panel B labels AF is A3F - and with AF added
# at its true position, hard against the abscissa, since that is the point of
# the correction.
ARROW = '#1f6fd0'


def trace_at(name, x_ms):
    return jump.P[BG.i(name)][int(np.searchsorted(t_ms, x_ms))]


def label(ax, text, x_text, y_text, name, x_anchor, mark=False, rad=0.0):
    y = trace_at(name, x_anchor)
    if mark:
        ax.plot([x_anchor], [y], marker='o', ms=3.5, color='black', zorder=6)
    ax.annotate(text, xy=(x_anchor, y), xytext=(x_text, y_text),
                fontsize=11, fontweight='bold', color='black',
                ha='center', va='center', zorder=6,
                arrowprops=dict(arrowstyle='->', color=ARROW, lw=1.4,
                                shrinkA=4, shrinkB=3,
                                connectionstyle='arc3,rad=%g' % rad))


axA.annotate(r'$P_{\rm open}$',
             xy=(22.0, jump.Popen[int(np.searchsorted(t_ms, 22.0))]),
             xytext=(29.0, 0.86), fontsize=11, fontweight='bold', color='black',
             ha='center', va='center', zorder=6,
             arrowprops=dict(arrowstyle='->', color=ARROW, lw=1.4,
                             shrinkA=4, shrinkB=3))
label(axA, 'A3F*', 13.5, 0.56, 'A3F*', 18.5)
label(axA, 'A2F*', 12.0, 0.29, 'A2F*', 18.0)
label(axA, 'R',    35.5, 0.50, 'R',    30.0)
```

```
label(axA, 'AR',   33.0, 0.31, 'AR',   27.0)
label(axA, 'A2R',  38.0, 0.19, 'A2R',  30.0)

label(axB, 'A3F',  10.5, 0.079, 'A3F',  12.5)
label(axB, 'A2F*', 11.5, 0.033, 'A2F*', 12.5)
label(axB, 'A2R',  24.0, 0.078, 'A2R',  20.0)
label(axB, 'A2F',  23.0, 0.031, 'A2F',  25.0)
label(axB, 'AF',   31.0, 0.036, 'AF',   28.0, mark=True, rad=0.18)

fig.suptitle('Figure 6- occupancies during a 0 → 1 mM → 0 glycine jump ␣
 ↪[bgtv2a]')
fig.tight_layout(rect=[0.02, 0.01, 1, 0.97])
plt.show()
```

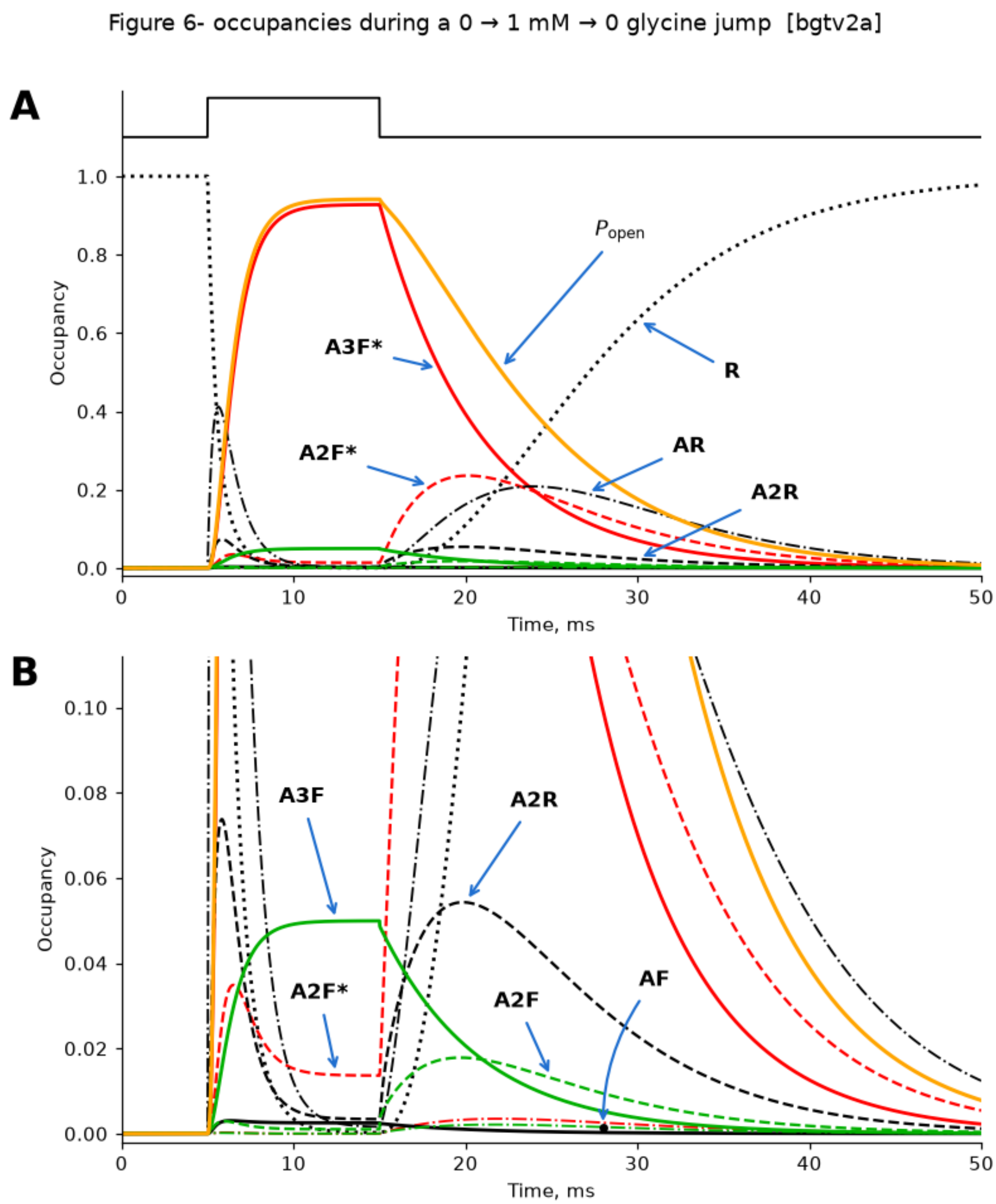

7.1 The numbers quoted in the Fig. 6 text

> *"Diliganded openings (A_2F^*, red dashed line) rise to a peak of around 3.5 % during the pulse, and then decline as a third ligand is bound. During offset, diliganded openings rise to a peak of over 20 % and in the later stages of decay there are more diliganded than triliganded channels."*

[18]:
```
on = (jump.t > PREPULSE) & (jump.t <= PREPULSE + WIDTH)
off = jump.t > PREPULSE + WIDTH

def peak(name, mask):
    y = jump.P[BG.i(name)][mask]
    i = int(np.argmax(y))
    return y[i], t_ms[mask][i]

pk_on, t_on = peak('A2F*', on)
pk_off, t_off = peak('A2F*', off)
late = jump.t > 35e-3

print('A2F* peak during the pulse  : %6.3f %% at %5.2f ms   paper "around 3.
↪5%%"'
      % (100*pk_on, t_on))
print('A2F* peak during the offset : %6.3f %% at %5.2f ms   paper "over 20%%"'
      % (100*pk_off, t_off))
print('Popen plateau               : %6.4f' % jump.Popen[on].max())
print('more diliganded than triliganded open channels after 35 ms? %s'
      % bool(np.all(jump.P[BG.i('A2F*')][late] > jump.P[BG.i('A3F*')][late])))
```

```
A2F* peak during the pulse  :  3.488 % at  6.50 ms   paper "around 3.5%"
A2F* peak during the offset : 23.595 % at 20.15 ms   paper "over 20%"
Popen plateau               : 0.9412
more diliganded than triliganded open channels after 35 ms? True
```

The plateau at 0.05 during the pulse is `A3F` (Table 1: 4.99 %).

> **Two traces are interchanged in the published panel B.** The trace labelled `AF` there is in fact `A3F`, and vice versa; the line styles went with the labels rather than with the traces, so the plateau is also drawn dash-dot although the legend assigns solid lines to triliganded states.
>
> The identification is not marginal. The cell below gives the two occupancies 12 ms into the pulse, and Table 1 gives the corresponding equilibrium values at 1 mM: `A3F` = 4.99 %, `AF` = 0.0007 %. A trace at 0.05 can only be `A3F`.
>
> This is the subject of a corrigendum to the paper.

[19]:
```
i12 = int(np.searchsorted(t_ms, 12.0))
for n in ('A3F', 'A2F', 'AF'):
    print('p(%-4s) at t = 12 ms = %.7f' % (n, jump.P[BG.i(n)][i12]))
```

```
p(A3F ) at t = 12 ms = 0.0498142
p(A2F ) at t = 12 ms = 0.0010377
p(AF  ) at t = 12 ms = 0.0000077
```

8 Mechanism diagram helper (used for Figures 7 and 8)

Figures 7 and 8 are the mechanism of Fig. 3 with a number written against every arrow. The helper below lays the ten states out as in Fig. 3- resting on top, flipped in the middle, open at the bottom- and labels each of the 22 directed transitions.

[20]:

```
# The mechanism is drawn the way the paper draws it in Figs. 3, 7 and 8: the
# states on a grid, one row per conformation, joined by closely spaced pairs of
# harpoon arrows with a number written against each direction.
#
# Two details make it look like the published figure. The arrowheads are
# harpoons - a single long, shallow barb swept back from the tip - rather than
# ordinary triangles; and the barb always falls on the OUTSIDE of the pair,
# which comes out automatically by putting it on the left of the direction of
# travel. The two arrows also sit close together, about one line width apart,
# so a step reads as one reversible transition rather than two arrows.

COL_SPACING = 1.4
ROW_SPACING = 1.4                       # equal spacing gives a square grid

PAIR_OFFSET = 0.026 * COL_SPACING       # half the gap between the two arrows
LABEL_GAP   = 0.30 * COL_SPACING        # clearance left around a state label
HEAD_LENGTH = 0.20 * COL_SPACING
HEAD_WIDTH  = 0.046 * COL_SPACING
LINE_WIDTH  = 1.1

GRID = {   # state -> (column, row, the paper's state number)
    'R':   (0, 2, 10), 'AR':   (1, 2, 9), 'A2R':  (2, 2, 8), 'A3R':  (3, 2, 7),
    'AF':  (1, 1, 6),  'A2F':  (2, 1, 5), 'A3F':  (3, 1, 4),
    'AF*': (1, 0, 1),  'A2F*': (2, 0, 2), 'A3F*': (3, 0, 3),
}
LAYOUT = {name: (col * COL_SPACING, row * ROW_SPACING, number)
          for name, (col, row, number) in GRID.items()}

# Eleven connections, not thirteen: binding along the resting row and the
# flipped row, flipping between them, opening below. The open states do not
# interconvert by binding, so the bottom row has no horizontal steps.
EDGES = [('R', 'AR'), ('AR', 'A2R'), ('A2R', 'A3R'),
         ('AF', 'A2F'), ('A2F', 'A3F'),
         ('AR', 'AF'), ('A2R', 'A2F'), ('A3R', 'A3F'),
         ('AF', 'AF*'), ('A2F', 'A2F*'), ('A3F', 'A3F*')]

# one mathtext string per label, so subscripts and the asterisk sit tight
SLAB = {'R': r'$\mathrm{R}$',        'AR': r'$\mathrm{AR}$',
        'A2R': r'$\mathrm{A_2R}$',   'A3R': r'$\mathrm{A_3R}$',
        'AF': r'$\mathrm{AF}$',      'A2F': r'$\mathrm{A_2F}$',
        'A3F': r'$\mathrm{A_3F}$',   'AF*': r'$\mathrm{AF^*}$',
        'A2F*': r'$\mathrm{A_2F^*}$', 'A3F*': r'$\mathrm{A_3F^*}$'}
```

```python
RESTING, FLIPPED, OPEN = 'black', '#00a651', '#ed1c24'
ROWCOL = {2 * ROW_SPACING: RESTING, ROW_SPACING: FLIPPED, 0.0: OPEN}
STATE_NUMBER_COLOUR = '#1c9ad6'


def harpoon(ax, start, end):
    """A line with one barb swept back from the tip, on its left."""
    (x0, y0), (x1, y1) = start, end
    dx, dy = x1 - x0, y1 - y0
    length = (dx * dx + dy * dy) ** 0.5
    ux, uy = dx / length, dy / length          # along the arrow
    nx, ny = -uy, ux                           # to its left

    ax.plot([x0, x1], [y0, y1], color='black', lw=LINE_WIDTH,
            solid_capstyle='butt', zorder=2)

    bx, by = x1 - ux * HEAD_LENGTH, y1 - uy * HEAD_LENGTH
    barb = [(x1, y1),
            (bx + nx * HEAD_WIDTH, by + ny * HEAD_WIDTH),
            (x1 - ux * HEAD_LENGTH * 0.40, y1 - uy * HEAD_LENGTH * 0.40)]
    ax.add_patch(plt.Polygon(barb, closed=True, facecolor='black',
                             edgecolor='none', zorder=3))


def fmt3(v):
    """Format a value the way the published figures print them: plain decimal,
    no exponent, trailing zeros stripped, 1.0 as a bare 1. Frequencies reach
    thousands per second and are printed whole, so f43 comes out as 6472."""
    if v == 0:
        return '0'
    if abs(v) >= 1000:
        return '%.0f' % v
    s = '%.3g' % v
    if 'e' in s:                               # values below 1e-4
        s = '%.7f' % v
    if '.' in s:
        s = s.rstrip('0').rstrip('.')
    return s


def draw_mechanism(M, model, panel='', caption='', fmt_fn=fmt3, ax=None,
                   figsize=(7.6, 4.7)):
    """Draw the flip mechanism, labelling each directed edge i->j with M[i, j].
↪"""
    if ax is None:
        _, ax = plt.subplots(figsize=figsize)
```

```
for a, b in EDGES:
    xa, ya, _ = LAYOUT[a]
    xb, yb, _ = LAYOUT[b]

    if ya == yb:                                    # horizontal pair
        forward, back = (a, b) if xa < xb else (b, a)
        left, right = min(xa, xb) + LABEL_GAP, max(xa, xb) - LABEL_GAP
        span = right - left

        harpoon(ax, (left, ya + PAIR_OFFSET), (right, ya + PAIR_OFFSET))
        harpoon(ax, (right, ya - PAIR_OFFSET), (left, ya - PAIR_OFFSET))

        ax.text(left + 0.20 * span, ya + PAIR_OFFSET + 0.09,
                fmt_fn(M[model.i(forward), model.i(back)]),
                ha='center', va='bottom', fontsize=9)
        ax.text(left + 0.78 * span, ya - PAIR_OFFSET - 0.09,
                fmt_fn(M[model.i(back), model.i(forward)]),
                ha='center', va='top', fontsize=9)
    else:                                           # vertical pair
        upper, lower = (a, b) if ya > yb else (b, a)
        top, bottom = max(ya, yb) - LABEL_GAP, min(ya, yb) + LABEL_GAP

        harpoon(ax, (xa + PAIR_OFFSET, top), (xa + PAIR_OFFSET, bottom))
        harpoon(ax, (xa - PAIR_OFFSET, bottom), (xa - PAIR_OFFSET, top))

        ax.text(xa + PAIR_OFFSET + 0.10, top - 0.02,
                fmt_fn(M[model.i(upper), model.i(lower)]),
                ha='left', va='top', fontsize=9)
        ax.text(xa - PAIR_OFFSET - 0.10, bottom + 0.02,
                fmt_fn(M[model.i(lower), model.i(upper)]),
                ha='right', va='bottom', fontsize=9)

for name, (x, y, pno) in LAYOUT.items():
    ax.text(x, y, SLAB[name], ha='center', va='center', fontsize=19,
            color=ROWCOL[y])
    ax.text(x + 0.13, y - 0.11, str(pno), ha='left', va='top',
            fontsize=9, color=STATE_NUMBER_COLOUR)

top_row, left_col = 2 * ROW_SPACING, 0.0
if panel:
    ax.text(left_col - 1.30, top_row + 0.50, panel, fontsize=20,
            fontweight='bold', ha='left', va='center')
if caption:
    ax.text(left_col - 0.80, ROW_SPACING, caption, fontsize=14,
            ha='center', va='center')
```

```
    # equal aspect, or the harpoons shear and the pairs stop looking parallel
    ax.set_aspect('equal')
    ax.set_xlim(left_col - 1.35, 3 * COL_SPACING + 0.45)
    ax.set_ylim(-0.50, top_row + 0.75)
    ax.axis('off')
    return ax
```

9 Figure 7- transition probabilities

The probability that the next transition out of state i, whenever it happens, goes to state j (paper Eq. 2; Colquhoun & Hawkes 1982, Eq. 1.5):

$$\pi_{ij} = \frac{q_{ij}}{\sum_{j \neq i} q_{ij}} = -\frac{q_{ij}}{q_{ii}}.$$

These are independent of time and do **not** require equilibrium, but they do depend on concentration. The probabilities on all arrows leaving a state sum to 1.

[21]:
```
PI = {c: transition_probability(BG.Q(c)) for c in (C_LOW, C_HIGH)}
for c, tag in ((C_LOW, '10 uM'), (C_HIGH, '1 mM')):
    rs = PI[c].sum(axis=1)
    print('%-6s : row sums min %.10f  max %.10f  (must be 1)' % (tag, rs.min(),␣
↪rs.max()))
```

```
10 uM  : row sums min 1.0000000000  max 1.0000000000  (must be 1)
1 mM   : row sums min 1.0000000000  max 1.0000000000  (must be 1)
```

[22]:
```
fig, axes = plt.subplots(2, 1, figsize=(7.6, 9.4))
draw_mechanism(PI[C_LOW],  BG, 'A', '10 µM\nglycine', ax=axes[0])
draw_mechanism(PI[C_HIGH], BG, 'B', '1 mM\nglycine',  ax=axes[1])
fig.suptitle('Figure 7- transition probabilities $\\pi_{ij}$   [bgtv2a]',
             fontsize=13, y=0.965)
fig.tight_layout(rect=[0, 0, 1, 0.95])
plt.show()
```

Figure 7- transition probabilities π_{ij} [bgtv2a]

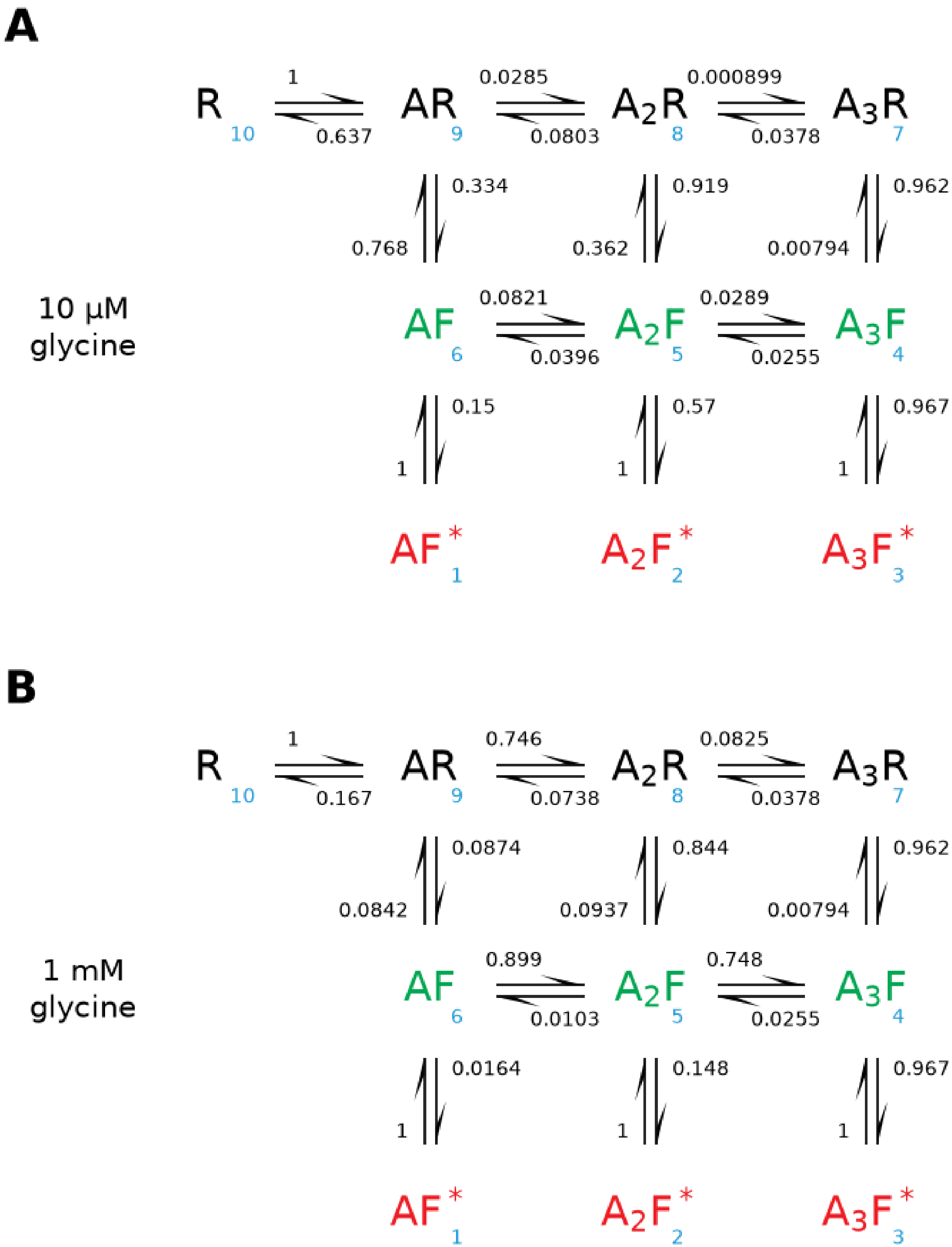

[23]:
```
def pi(c, a, b):
    return PI[c][BG.i(a), BG.i(b)]

print('pi(R -> AR) = %.6f at 10 uM and %.6f at 1 mM'
      % (pi(C_LOW, 'R', 'AR'), pi(C_HIGH, 'R', 'AR')))
print('  -> exactly 1, because binding is the only way to leave state 10.')
```

```
pi(R -> AR) = 1.000000 at 10 uM and 1.000000 at 1 mM
  -> exactly 1, because binding is the only way to leave state 10.
```

10 Figure 8- transition frequencies at equilibrium

The number of $i \to j$ transitions per second (paper Eq. 3):

$$f_{ij} = p_i(t)\, q_{ij},$$

evaluated here with the equilibrium occupancies $p(\infty)$. Because the mechanism obeys microscopic reversibility, $f_{ij} = f_{ji}$ at equilibrium- a useful check on the arithmetic, and one that `bgtv2a` passes to machine precision.

[24]:
```
FREQ = {c: transition_frequency(BG.Q(c), BG.pinf(c))
        for c in (C_LOW, C_HIGH)}
for c, tag in ((C_LOW, '10 uM'), (C_HIGH, '1 mM')):
    d = np.abs(FREQ[c] - FREQ[c].T).max()
    print('%-6s : max |f_ij - f_ji| = %.3e   (%.1e relative)'
          % (tag, d, d / np.abs(FREQ[c]).max()))
print()
print('For comparison, the Fig 3 averaged rates give:')
for c, tag in ((C_LOW, '10 uM'), (C_HIGH, '1 mM')):
    Fa = transition_frequency(AV.Q(c), AV.pinf(c))
    d = np.abs(Fa - Fa.T).max()
    print('%-6s : max |f_ij - f_ji| = %.3e   (%.1e relative)'
          % (tag, d, d / np.abs(Fa).max()))
```

```
10 uM  : max |f_ij - f_ji| = 5.626e-08   (2.1e-09 relative)
1 mM   : max |f_ij - f_ji| = 3.407e-08   (5.3e-12 relative)

For comparison, the Fig 3 averaged rates give:
10 uM  : max |f_ij - f_ji| = 1.584e-02   (7.0e-04 relative)
1 mM   : max |f_ij - f_ji| = 5.687e-02   (8.8e-06 relative)
```

[25]:
```
fig, axes = plt.subplots(2, 1, figsize=(7.6, 9.4))
draw_mechanism(FREQ[C_LOW],  BG, 'A', '10 µM\nglycine', ax=axes[0])
draw_mechanism(FREQ[C_HIGH], BG, 'B', '1 mM\nglycine',  ax=axes[1])
fig.suptitle('Figure 8- transition frequencies at equilibrium (s$^{-1}$)  ␣
↪[bgtv2a]',
             fontsize=13, y=0.965)
```

```
fig.tight_layout(rect=[0, 0, 1, 0.95])
plt.show()
```

Figure 8- transition frequencies at equilibrium (s^{-1}) [bgtv2a]

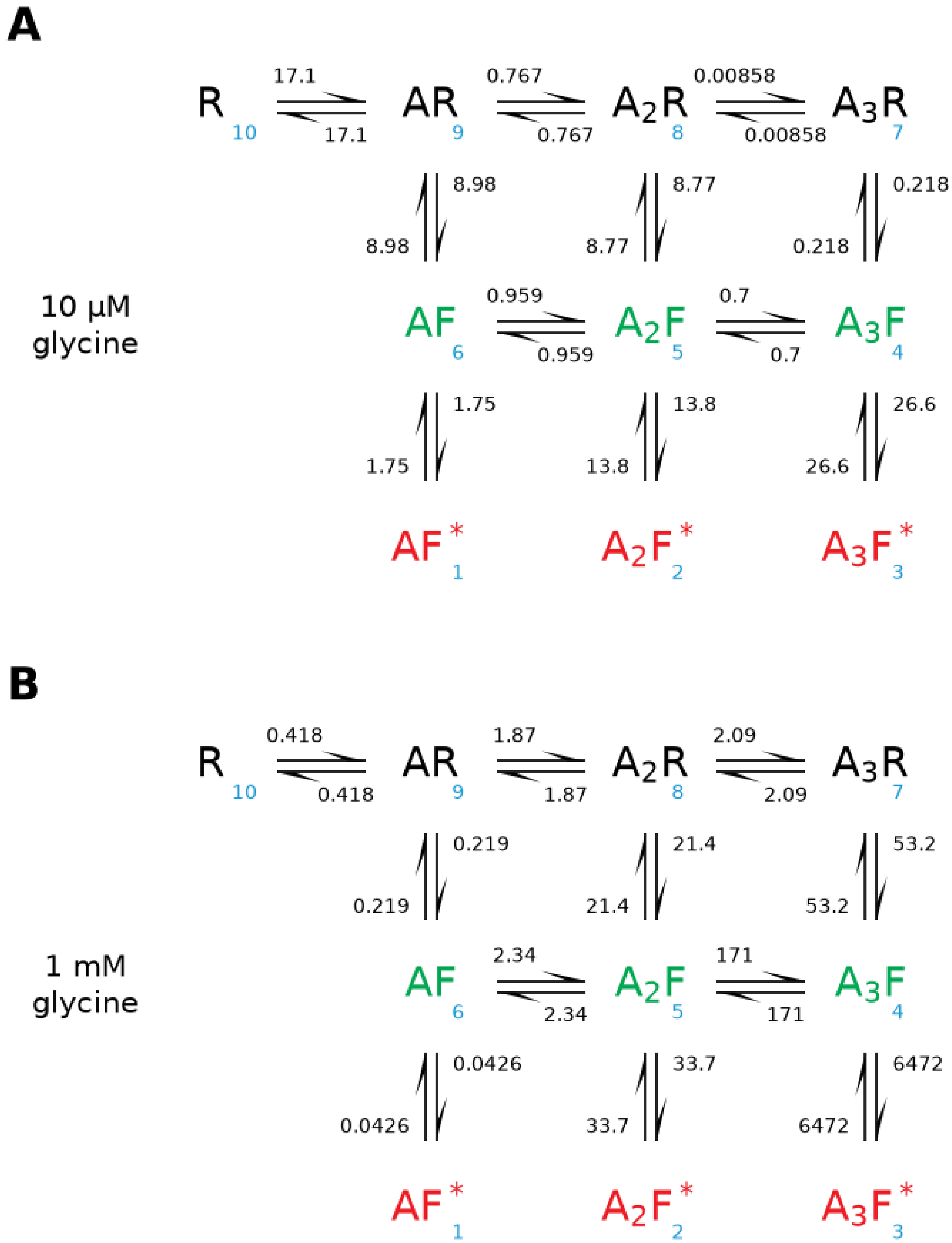

10.1 The frequencies quoted in the text

> *"At 1 mM glycine … by far the most frequent transition at equilibrium is between the fully liganded flipped state and the open state, $f_{43} = f_{34} = 6{,}472$ transitions per second … In contrast, the first occupancy of the resting state, $f_{10,9}$ is rare, only 0.418 per second … the initial frequency of bindings is thus 1,850 bindings per second at $t = 0$, and it falls to 0.418 per second at equilibrium."*

[26]:
```
def f_(c, a, b):
    return FREQ[c][BG.i(a), BG.i(b)]

q109 = BG.Q(C_HIGH)[BG.i('R'), BG.i('AR')]     # = 3*k(+1)*c
chk = pd.DataFrame([
    ('f43 = f(A3F -> A3F*)', f_(C_HIGH, 'A3F', 'A3F*'), 6472,  's^-1'),
    ('f34 = f(A3F* -> A3F)', f_(C_HIGH, 'A3F*', 'A3F'), 6472,  's^-1'),
    ('f10,9 = f(R -> AR)',    f_(C_HIGH, 'R', 'AR'),     0.418, 's^-1'),
    ('f9,10 = f(AR -> R)',    f_(C_HIGH, 'AR', 'R'),     0.418, 's^-1'),
    ('initial binding rate p10(0)*q10,9 at t=0', q109,  1850,  's^-1'),
], columns=['quantity', 'bgtv2a', 'published', 'units'])
chk['% diff'] = 100 * (chk['bgtv2a'] / chk['published'] - 1)
show(chk.style.format({'bgtv2a': '{:.6g}', 'published': '{:.6g}', '% diff': '{:
 ↪+.3f}'}))
```

	quantity	bgtv2a	published	units	% diff
0	f43 = f(A3F -> A3F*)	6471.73	6472	s^-1	-0.004
1	f34 = f(A3F* -> A3F)	6471.73	6472	s^-1	-0.004
2	f10,9 = f(R -> AR)	0.417522	0.418	s^-1	-0.114
3	f9,10 = f(AR -> R)	0.417522	0.418	s^-1	-0.114
4	initial binding rate p10(0)*q10,9 at t=0	1850.57	1850	s^-1	+0.031

[27]:
```
print('initial binding frequency = %.1f s^-1 = %.3f ms^-1 = %.5f us^-1'
      % (q109, q109*1e-3, q109*1e-6))
print('at equilibrium            = %.4f s^-1' % f_(C_HIGH, 'R', 'AR'))
print('ratio = %.0f-fold' % (q109 / f_(C_HIGH, 'R', 'AR')))
print()
print('With 1000 channels, %.0f would associate per second and %.0f dissociate.'
      % (1000*f_(C_HIGH, 'R', 'AR'), 1000*f_(C_HIGH, 'AR', 'R')))
print('(the paper: "With 1,000 channels, 418 would associate per second, and␣
 ↪418')
print(' would dissociate")')
```

```
initial binding frequency = 1850.6 s^-1 = 1.851 ms^-1 = 0.00185 us^-1
at equilibrium            = 0.4175 s^-1
ratio = 4432-fold

With 1000 channels, 418 would associate per second and 418 dissociate.
(the paper: "With 1,000 channels, 418 would associate per second, and 418
 would dissociate")
```

11 Preferred routes through the mechanism

Using the transition probabilities of Fig. 7, the probability of a particular route from the vacant resting receptor R (state 10) to the fully liganded open state A_3F^* (state 3) is the product of the π_{ij} along it. Note $\pi_{10,9} = 1$, so it drops out.

Route	Path	Description
via 8→5	10→9→8→**5**→4→3	flips while **di**liganded
clockwise	10→9→8→**7**→4→3	binds all three, then flips
anticlockwise	10→9→**6**→5→4→3	flips while **mono**liganded

[28]:
```
ROUTES = {
    'via 8,5       10>9>8>5>4>3': [('AR','A2R'), ('A2R','A2F'), ('A2F','A3F'), ('A3F','A3F*')],
    'clockwise     10>9>8>7>4>3': [('AR','A2R'), ('A2R','A3R'), ('A3R','A3F'), ('A3F','A3F*')],
    'anticlockwise 10>9>6>5>4>3': [('AR','AF'),  ('AF','A2F'),  ('A2F','A3F'), ('A3F','A3F*')],
}
PUB_R = {'via 8,5       10>9>8>5>4>3': (7.3e-4, 0.455),
         'clockwise     10>9>8>7>4>3': (2.8e-5, 0.0573),
         'anticlockwise 10>9>6>5>4>3': (7.7e-4, 0.0568)}

rows = []
for name, steps in ROUTES.items():
    rows.append({
        'route': name,
        '10 uM bgtv2a': np.prod([pi(C_LOW, a, b) for a, b in steps]),
        '10 uM pub': PUB_R[name][0],
        '1 mM bgtv2a': np.prod([pi(C_HIGH, a, b) for a, b in steps]),
        '1 mM pub': PUB_R[name][1],
    })
routes = pd.DataFrame(rows).set_index('route')
show(routes.style.format('{:.4g}'))
```

route	10 uM bgtv2a	10 uM pub	1 mM bgtv2a	1 mM pub
via 8,5 10>9>8>5>4>3	0.0007315	0.00073	0.4552	0.455
clockwise 10>9>8>7>4>3	2.385e-05	2.8e-05	0.05727	0.0573
anticlockwise 10>9>6>5>4>3	0.0007654	0.00077	0.05685	0.0568

Five of the six route probabilities match the published values to the printed precision. The sixth does not, and the discrepancy looks like a misprint in the paper rather than a difference in the calculation:

> **The clockwise route at 10 µM.** The paper gives 2.8×10^{-5}; `bgtv2a` gives $\mathbf{2.385 \times 10^{-5}}$, which would print as 2.4×10^{-5}. The same expression, $\pi_{98}\pi_{87}\pi_{74}\pi_{43}$, reproduces

the 1 mM value (0.05727 against a published 0.0573) exactly, so the formula is not in doubt.

The paper's next sentence supports the computed value: it says the other two routes are "about 30 times more likely than the clockwise route". With 2.385×10^{-5} those ratios are 30.7 and 32.1; with 2.8×10^{-5} they would be 26.1 and 27.5, which would not have been described as about 30.

The paper's conclusions follow, and are unaffected:

- **At 1 mM** the dominant route flips while *di*liganded (10→9→8→5→4→3) and is about 8 times more probable than either route round the outside of the rectangle, which are nearly equal to each other.
- **At 10 µM** the anticlockwise route (flipping while *mono*liganded) and the route via states 8 and 5 are comparable, and both are ~30× more likely than the clockwise route via state 7.

[29]:
```
r1, r0 = routes['1 mM bgtv2a'], routes['10 uM bgtv2a']
print('At 1 mM  : dominant route is %.2f x clockwise and %.2f x anticlockwise'
      % (r1.iloc[0]/r1.iloc[1], r1.iloc[0]/r1.iloc[2]))
print('           paper: "about 8 times larger than ... either of the routes␣
↪round the outside"')
print('           clockwise / anticlockwise = %.3f  (paper: nearly equal)'
      % (r1.iloc[1]/r1.iloc[2]))
print()
print('At 10 uM : via-8,5 / clockwise = %.0f x ;  anticlockwise / clockwise = %.
↪0f x'
      % (r0.iloc[0]/r0.iloc[1], r0.iloc[2]/r0.iloc[1]))
print('           paper: "both are about 30 times more likely than the␣
↪clockwise route"')
```

```
At 1 mM  : dominant route is 7.95 x clockwise and 8.01 x anticlockwise
           paper: "about 8 times larger than … either of the routes round the
outside"
           clockwise / anticlockwise = 1.007  (paper: nearly equal)

At 10 uM : via-8,5 / clockwise = 31 x ;  anticlockwise / clockwise = 32 x
           paper: "both are about 30 times more likely than the clockwise route"
```

11.1 Conditional probabilities (Eqs 4 and 5)

The paper refines the calculation by noting that opening while mono- or diliganded is irrelevant to reaching A_3F^* by this route, so π_{54} should be replaced by the probability of a 5→4 transition *given that the channel does not open*, and likewise for π_{65}:

$$\frac{\pi_{54}}{1-\pi_{52}}, \qquad \frac{\pi_{65}}{1-\pi_{61}}.$$

The paper concludes these corrections are "sufficiently small that the conclusions about predominant route are not changed"- checked below by redoing the route ranking with the corrected probabilities.

[30]:
```
rows = []
for c, tag in ((C_LOW, '10 uM'), (C_HIGH, '1 mM')):
    rows.append({'c': tag,
                 'pi54': pi(c, 'A2F', 'A3F'),
                 'pi54/(1-pi52)': pi(c, 'A2F', 'A3F') / (1 - pi(c, 'A2F',␣
 ↪'A2F*')),
                 'pi65': pi(c, 'AF', 'A2F'),
                 'pi65/(1-pi61)': pi(c, 'AF', 'A2F') / (1 - pi(c, 'AF',␣
 ↪'AF*'))})
show(pd.DataFrame(rows).set_index('c').style.format('{:.5g}'))

print('Route probabilities using the conditional forms:')
for c, tag in ((C_LOW, '10 uM'), (C_HIGH, '1 mM')):
    p54c = pi(c, 'A2F', 'A3F') / (1 - pi(c, 'A2F', 'A2F*'))
    p65c = pi(c, 'AF', 'A2F') / (1 - pi(c, 'AF', 'AF*'))
    via85 = pi(c,'AR','A2R') * pi(c,'A2R','A2F') * p54c * pi(c,'A3F','A3F*')
    cw    = pi(c,'AR','A2R') * pi(c,'A2R','A3R') * pi(c,'A3R','A3F') *␣
 ↪pi(c,'A3F','A3F*')
    acw   = pi(c,'AR','AF')  * p65c             * p54c * pi(c,'A3F','A3F*')
    order = sorted([('via 8,5', via85), ('clockwise', cw), ('anticlockwise',␣
 ↪acw)],
                   key=lambda kv: -kv[1])
    print('  %-6s via8,5 %.4g   clockwise %.4g   anticlockwise %.4g   -> %s'
          % (tag, via85, cw, acw, ' > '.join(k for k, _ in order)))
```

c	pi54	pi54/(1-pi52)	pi65	pi65/(1-pi61)
10 uM	0.028877	0.067136	0.082059	0.096484
1 mM	0.74833	0.878	0.89939	0.91437

```
Route probabilities using the conditional forms:
  10 uM   via8,5 0.001701   clockwise 2.385e-05   anticlockwise 0.002092   ->
anticlockwise > via 8,5 > clockwise
  1 mM    via8,5 0.5341   clockwise 0.05727   anticlockwise 0.06781   -> via 8,5
> anticlockwise > clockwise
```

12 Summary- everything against the published values

[31]:
```
def summarise(m, jump_pk=None):
    Q = m.Q(C_HIGH)
    F = transition_frequency(Q, m.pinf(C_HIGH))
    P = {c: transition_probability(m.Q(c)) for c in (C_LOW, C_HIGH)}
    p = lambda c, a, b: P[c][m.i(a), m.i(b)]
    pl, ph = m.pinf(C_LOW), m.pinf(C_HIGH)
    d = {
        'Table 1: A3F* at 1 mM (%)':   100*ph[m.i('A3F*')],
```

```
        'Table 1: R at 10 uM (%)':     100*pl[m.i('R')],
        'Table 1: AR at 10 uM (%)':    100*pl[m.i('AR')],
        'Table 1: A3F at 1 mM (%)':    100*ph[m.i('A3F')],
        'Table 1: A2F* at 1 mM (%)':   100*ph[m.i('A2F*')],
        'Fig 8: f43 at 1 mM (s^-1)':   F[m.i('A3F'), m.i('A3F*')],
        'Fig 8: f10,9 at 1 mM (s^-1)': F[m.i('R'), m.i('AR')],
        'text: initial binding (s^-1)': Q[m.i('R'), m.i('AR')],
        'text: lifetime of R at 1 mM (ms)': -1e3 / Q[m.i('R'), m.i('R')],
    }
    for nm, steps in ROUTES.items():
        d['route %s at 1 mM' % nm.split()[0]] = np.prod(
            [p(C_HIGH, a, b) for a, b in steps])
    return d

PUB = {
    'Table 1: A3F* at 1 mM (%)': 92.77, 'Table 1: R at 10 uM (%)': 92.57,
    'Table 1: AR at 10 uM (%)': 6.21,   'Table 1: A3F at 1 mM (%)': 4.99,
    'Table 1: A2F* at 1 mM (%)': 1.36,
    'Fig 8: f43 at 1 mM (s^-1)': 6472,  'Fig 8: f10,9 at 1 mM (s^-1)': 0.418,
    'text: initial binding (s^-1)': 1850,
    'text: lifetime of R at 1 mM (ms)': 0.54,
    'route via at 1 mM': 0.455, 'route clockwise at 1 mM': 0.0573,
    'route anticlockwise at 1 mM': 0.0568,
}

summary = pd.DataFrame({'published': PUB, 'bgtv2a': summarise(BG),
                        'averaged': summarise(AV)})
summary['bgtv2a % err'] = 100 * (summary['bgtv2a'] / summary['published'] - 1)
summary['averaged % err'] = 100 * (summary['averaged'] / summary['published'] -␣
 ↪1)
show(
summary.style.format({'published': '{:.5g}', 'bgtv2a': '{:.5g}', 'averaged': '{:
 ↪.5g}',
                      'bgtv2a % err': '{:+.2f}', 'averaged % err': '{:+.2f}'}) \
       .background_gradient(cmap='RdYlGn_r', subset=['bgtv2a % err'], vmin=-20,␣
 ↪vmax=20) \
       .background_gradient(cmap='RdYlGn_r', subset=['averaged % err'],␣
 ↪vmin=-20, vmax=20))
```

	published	bgtv2a	averaged	bgtv2a % err	averaged % err
Table 1: A3F* at 1 mM (%)	92.77	92.775	92.525	+0.01	-0.26
Table 1: R at 10 uM (%)	92.57	92.567	93.362	-0.00	+0.86
Table 1: AR at 10 uM (%)	6.21	6.2147	5.5083	+0.08	-11.30
Table 1: A3F at 1 mM (%)	4.99	4.9932	5.0207	+0.06	+0.62
Table 1: A2F* at 1 mM (%)	1.36	1.3617	1.6071	+0.13	+18.17
Fig 8: f43 at 1 mM (s^-1)	6472	6471.7	6476.7	-0.00	+0.07
Fig 8: f10,9 at 1 mM (s^-1)	0.418	0.41752	0.4851	-0.11	+16.05
text: initial binding (s^-1)	1850	1850.6	1770	+0.03	-4.32
text: lifetime of R at 1 mM (ms)	0.54	0.54037	0.56497	+0.07	+4.62
route via at 1 mM	0.455	0.45522	0.44197	+0.05	-2.86
route clockwise at 1 mM	0.0573	0.057266	0.048627	-0.06	-15.14
route anticlockwise at 1 mM	0.0568	0.056848	0.071324	+0.08	+25.57

12.1 Reading the summary

With `bgtv2a` every quantity agrees with the paper to within the precision the paper prints- typically a few hundredths of a percent, and the residuals are consistent with the published values having been rounded to 3–4 significant figures rather than with any difference in the calculation.

The `averaged` column shows what the Fig. 3 mean rate constants give: fine for the dominant occupancies and for f_{43}, but 11–26 % out on the quantities that depend on the smallest occupancy ($p_R = 0.023$ %) or on the association rate, and it misorders the two outside routes at 1 mM. That column is a useful caution: reproducing a paper from the rate constants in a mechanism *figure* is not always the same as reproducing it from the rate constants actually used.

12.2 Where the two datasets came from

	source	microscopic reversibility
`bgtv2a.mec`	one fitted dataset, Burzomato et al. 2004	exact
`GlyR_flip`	mean of three datasets, as drawn in Fig. 3	3–4 % off

13 Automated verification

The cell below raises if any of the published values stops being reproduced, so running this notebook non-interactively

`jupyter nbconvert --to notebook --execute ColquhounLape2012_reproduction.ipynb`

is a regression test on the paper's numbers, not merely a check that the code runs. This is what continuous integration executes.

[32]:
```
TOL = 0.25            # per cent; the largest residual actually observed is 0.13 %

# 1. every published value, against the paper
bad = summary.index[abs(summary['bgtv2a % err']) > TOL].tolist()
assert not bad, 'no longer reproduced to %.2f %%: %s' % (TOL, bad)
print('%d published values reproduced to within %.2f %%   (max |err| = %.3f %%)'
```

```
      % (len(summary), TOL, summary['bgtv2a % err'].abs().max()))

# 2. the Fig. 6 B identification: the plateau at ~0.05 is A3F, not AF
p_a3f = jump.P[BG.i('A3F')][i12]
p_af = jump.P[BG.i('AF')][i12]
assert p_a3f > 0.04, 'A3F plateau missing: %g' % p_a3f
assert p_af < 1e-5, 'AF unexpectedly large: %g' % p_af
print('Fig 6B at t = 12 ms:  A3F = %.5f   AF = %.7f   ratio = %.0f'
      % (p_a3f, p_af, p_a3f / p_af))

# 3. the embedded rate constants obey microscopic reversibility
for c, tag in ((C_LOW, '10 uM'), (C_HIGH, '1 mM')):
    Fm = transition_frequency(BG.Q(c), BG.pinf(c))
    rel = np.abs(Fm - Fm.T).max() / np.abs(Fm).max()
    assert rel < 1e-6, 'reversibility violated at %s: %g' % (tag, rel)
    print('microscopic reversibility at %-6s: %.2e relative' % (tag, rel))

# 4. the embedded matrix is a proper Q matrix
for c, tag in ((C_LOW, '10 uM'), (C_HIGH, '1 mM')):
    assert np.abs(BG.Q(c).sum(axis=1)).max() < 1e-6
    assert abs(BG.pinf(c).sum() - 1.0) < 1e-9
print('\nALL CHECKS PASSED')
```

```
12 published values reproduced to within 0.25 %   (max |err| = 0.129 %)
Fig 6B at t = 12 ms:  A3F = 0.04981   AF = 0.0000077   ratio = 6445
microscopic reversibility at 10 uM : 2.12e-09 relative
microscopic reversibility at 1 mM  : 5.26e-12 relative

ALL CHECKS PASSED
```
